# Removal of metals and emergent contaminants from liquid digestates in constructed wetlands for agricultural reuse

**DOI:** 10.3389/fmicb.2024.1388895

**Published:** 2024-06-06

**Authors:** Pau Porras-Socias, Maria Paola Tomasino, Joana P. Fernandes, Alexandre B. De Menezes, Belén Fernández, Gavin Collins, Maria João Alves, Ricardo Castro, Carlos R. Gomes, C. Marisa R. Almeida, Ana Paula Mucha

**Affiliations:** ^1^CIIMAR, Interdisciplinary Centre of Marine and Environmental Research, University of Porto, Matosinhos, Portugal; ^2^Chemistry and Biochemistry Department, Faculty of Sciences, University of Porto, Porto, Portugal; ^3^Microbiology, School of Biological and Chemical Sciences and Ryan Institute, University of Galway, Galway, Ireland; ^4^Sustainability in Biosystems Programme, IRTA, Institute of Agrifood Research and Technology, Caldes de Montbui, Spain; ^5^TratoLixo—Tratamento de Resíduos Sólidos, E.I.M. S.A., São Domingos de Rana, Portugal; ^6^Biology Department, Faculty of Sciences, University of Porto, Porto, Portugal

**Keywords:** constructed wetlands, metals, antibiotics, antibiotic resistance genes, anaerobic digestion effluent, *Sparganium erectum*

## Abstract

Given the increasing pressure on water bodies, it is imperative to explore sustainable methodologies for wastewater treatment and reuse. The simultaneous presence of multiples contaminants in complex wastewater, such as the liquid effluents from biogas plants, can compromise biological treatment effectiveness for reclaiming water. Vertical subsurface flow constructed wetlands were established as low-cost decentralized wastewater treatment technologies to treat the liquid fraction of digestate from municipal organic waste with metals, antibiotics, and antibiotic resistance genes, to allow its reuse in irrigation. Twelve lab-scale planted constructed wetlands were assembled with gravel, light expanded clay aggregate and sand, testing four different treating conditions (liquid digestate spiked with oxytetracycline, sulfadiazine, or ofloxacin, at 100 μg/ L, or without dosing) during 3 months. Physicochemical parameters (pH, chemical oxygen demand (COD), nutrients, metals, and antibiotics), the microbial communities dynamics (through 16S high-throughput sequencing) and antibiotic resistance genes removal (qPCR) were monitored in influents and effluents. Systems removed 85.8%–96.9% of organic matter (as COD), over 98.1% of ammonium and phosphate ions, and 69.3%–99.4% of nitrate and nitrite ions, with no significant differences between the presence or absence of antibiotics. Removal of Fe, Mn, Zn, Cu, Pb and Cr exceeded 82% in all treatment cycles. The treatment also removed oxytetracycline, sulfadiazine and ofloxacin over 99%, and decreased *intl1, tetA, tetW*, *sul1* and *qnrS* gene copies. Nonetheless, after 3 months of ofloxacin dosing, *qnrS* gene started being detected. Removal processes relied on high HRT (14 days) and various mechanisms including sorption, biodegradation, and precipitation. Microbial community diversity in liquid digestate changed significantly after treatment in constructed wetlands with a decrease in the initial Firmicutes dominance, but with no clear effect of antibiotics on the microbial community structure. Removals above 85% and 94% were observed for *Streptococcus* and *Clostridium,* respectively. Results suggest that vertical subsurface flow constructed wetlands were a suitable technology for treating the liquid digestate to reuse it in irrigation agricultural systems, contributing to the circular bioeconomy concept. However, a more profound understanding of effective wastewater treatment strategies is needed to avoid antibiotic resistance genes dissemination.

## 1 Introduction

The transition towards circular economy is one of the challenges of the 21st century. In 2018, in response to the Circular Economy Action Plan, the European Commission created the Bioeconomy Strategy addressing, in part, the sustainable management of organic waste (European Commission, 2022b). About 60 million out of 138 million tons of municipal and industrial organic waste were valorised in the European Union in 2019 (Gilbert and Siebert, 2022), but the European Commission targets to increase the reusing strategies by 2035 (European Environment Agency, 2020). Anaerobic digestion is the most promising valorisation process of organic waste, generating bioenergy products and digestate. Digestate is a complex matrix of biosolids rich in organic matter, macro, and micronutrients, which makes it a potentially excellent fertilizer (European Environment Agency, 2020). However, most biogas plant practices prioritize the improvement of biogas production over the digestate management (Logan and Visvanathan, 2019). The management options of the digestate include in most cases the separation of the liquid and solid fractions, for the subsequent composting of the solid fraction for its use as a soil amendment (Wang et al., 2023). Processes to valorise the liquid fraction of the digestate (LFD) include membrane filtration, struvite precipitation, ammonia stripping, and microalgae cultivation. However, challenges such as high power consumption and maintenance costs of the processes, and early-development stages technologies, hinder its reuse and hence, LFD are frequently disposed in centralized wastewater treatment facilities (Lamolinara et al., 2022).

The LFD contains over 90% of water and represents above 80% of the total digestate weight. It comprises a high concentration of organic matter, soluble ions, namely ammonium, potassium and phosphate ions, and humic substances (Akhiar et al., 2017). Unfortunately, as many feedstocks contain chemical and biological pollutants that are not efficiently degraded during the anaerobic digestion process, LFD can harbor more complex pollutants namely metals (Dragicevic et al., 2018), organic contaminants, pathogens (Bloem et al., 2017), and other micropollutants (Venegas et al., 2021). For example, veterinary antibiotics, namely oxytetracycline, sulfadiazine and ofloxacin, among many others, have low removal rates during anaerobic digestion and are persistently found in LFD of reactors treating livestock manure in concentrations between 3.8 and 940 μg/L (Gurmessa et al., 2020; Yang et al., 2022). After assessing the risk of oxytetracycline, sulfadiazine and ofloxacin in water systems, these compounds were classified as high-risk (Ilyas et al., 2020). Antibiotics, in combination with metals, exert selective pressure on microorganisms and mobile genetic elements (MGEs) can promote the horizontal gene transfer of antibiotic resistance genes (ARGs; Wolak et al., 2023). Although ARGs can present 51% reduction along anaerobic digestion, many different ARGs and their associated antibiotic-resistant bacteria are persistent in the digestate (Goulas et al., 2020).

Water is a limited valuable resource, and as many other parts of the world, the European Union is suffering from growing pressure on water resources resulting from variable availability, climate change and poor water quality. Consequently, reusing reclaimed water is a crucial practice for efficient water resource management, ensuring a predictable water supply and reducing freshwater consumption (Chen et al., 2021). The agricultural sector is particularly interested in the reuse of the LFD for irrigation purposes in the fields, as a non-conventional water reuse source (Chen et al., 2021), since 71.7% of the total water withdrawal is used in agriculture (Food and Agriculture Organization of the United Nations, 2023). The Water Reuse Regulation establishes minimum water quality standards for the safe reuse of treated wastewater in agricultural irrigation (European Commission, 2020, 2022a). Hence, effective treatment of LFD is crucial before its reuse to avoid risks to the groundwaters and human health associated to the release of the LFD contaminants into the environment. Due to the high operation and maintenance costs of conventional wastewater treatments, there is growing interest in considering low-cost nature-based solutions for efficient wastewater treatment (Cross et al., 2021).

Constructed wetlands (CWs) are nature-based solutions that effectively treat wastewater through different physical, chemical, and biological reactions (Gorito et al., 2017). CWs, consisting of assemblages of substrates, vegetation and associated microorganisms, have low operation and maintenance requirements and show consistent performance to fluctuations in inputs, being primary, secondary, or tertiary treatment of a wide range of wastewater (Dotro et al., 2017). Previous works reported that CWs could successfully remove metals (Dias et al., 2020) and antibiotics in some wastewater types (Ilyas et al., 2020), being an efficient and fit-for-purpose treatment technology, However, a lingering risk remains as our understanding of CWs’ effectiveness in eliminating ARGs is still limited (Ilyas et al., 2020). Moreover, although CWs can be an option to reduce the organic matter and nutrient loads from LFD (Comino et al., 2013; Guo et al., 2016; Maucieri et al., 2016; Wu et al., 2016b), no research has been published on the removal of contaminants of emerging concern from this complex matrix of biosolids. CWs could fail to remove simultaneously a wide range of contaminants. To the best of our knowledge, this is the first study to focus on the potential of CWs to treat LFD for agricultural reuse, considering both chemical and biological contaminants, including metals, antibiotics, ARGs and potential pathogens.

This study aims to evaluate the potential of a new configuration of vertical subsurface flow CWs, at a microcosm scale, to remove simultaneously different types of pollutants from the LFD to allow its use in irrigation. Firstly, removals of organic matter and nutrients, and pH stabilization were assessed to confirm that CWs were treating the high load of organic matter, nitrogen, and phosphorus of the LFD. Secondly, the concentration of metals, antibiotics (oxytetracycline, sulfadiazine, and ofloxacin) and MGEs (*intI1*) and ARGs (*tetA, tetW, sul1* and *qnrS*) was analyzed in CWs influents and effluents to evaluate if the effluent was safe for water reuse. Third, microbial communities in the influent and effluent were characterized to monitor population shifts and structure and assess the removal of potentially pathogenic microorganisms.

## 2 Materials and methods

### 2.1 CWs assembly and acclimation

Twelve laboratory scale vertical subsurface flow CWs systems were assembled on 12th July 2021, each in 0.4 × 0.3 × 0.3 m^3^ plastic containers with a bottom layer of gravel (3 cm), a second layer of light expanded clay aggregate (LECA, 3 cm) and a top layer of sand (20 cm) in which *Sparganium erectum* plants were transplanted (Supplementary Figure S1). To our knowledge, it is the first time this plant species is used in this CWs design. *S. erectum* were harvested in the Ribeira da Certagem, Lavra, Portugal (N 41°15′31.252″; W 8°43′24.924″) on 11th July 2021, and were rinsed with abundant deionized water in the lab before the assembly. Each microcosm had between 3 and 4 individual plants, with a total fresh weight of around 700 g. The containers were wrapped with aluminum foil to prevent the photodegradation of compounds in the substrate and were placed in a greenhouse in the gardens of the Faculty of Sciences of the University of Porto (Portugal), under a natural light/dark regime with a temperature fluctuation between 14.0°C and 44.1°C in July 2021, and 2.0°C and 36.5°C in November 2021.

The systems simulated vertical subsurface flow CWs with the influent being poured on the surface and drained through the substrate layers of the systems. For the acclimation of the systems, each microcosm was saturated with 5.4 L of Hoagland nutrient solution. The effluent was daily recirculated and every 2/3 days, the nutrient solution was replaced with a new one. After 2 weeks, the solution was drained from CWs, and a three-step adaptation process started by adding LFD to the systems. Firstly, 1 L of a 1/10 dilution (v/v) with deionized water of the LFD was added to each system, recirculated for 7 days, and then removed. Secondly, new fresh LFD was added to each system, this time, 1 L of 1/4 dilution (v/v), being recirculated for 14 days, and removed. Thirdly, 1 L of 1/2 dilution (v/v) of the LFD was added to microcosms, recirculating it for 14 days, before removing it all. Deionized water was added to saturate the systems whenever necessary (filling the systems just below the surface to compensate evapotranspiration).

### 2.2 CWs experiments

The LFD was collected from TratoLixo, Mafra, Portugal (N 38°56′14.435″; W 9°17′5.3916″), a full-scale anaerobic digestion plant treating the organic fraction of municipal solid waste, processing 65,000 tons of organic waste annually from an intervention area with 100,000 inhabitants. LFD collection was performed every 14 days. The physicochemical characterization of the LFD was performed by the biogas plant company, except for chemical oxygen demand (COD) analysis.

Before adding the LFD to the systems, a homogenized 1/4 dilution (v/v) of the collected effluent with deionized water was prepared and allowed to stand overnight at room temperature to allow solids to settle. This dilution was chosen to avoid clogging of the CWs systems due to the high amount of dissolved solids, considering the acclimation results. The supernatant was then transferred to a clean vessel, where it was spiked with oxytetracycline, sulfadiazine, and ofloxacin methanolic solutions, or without dosing (control) to have a total of four different LFD to be treated in parallel (Supplementary Figure S1). C systems were treating 1 L of LFD without antibiotic spiking. OX, SD and OF CWs were the systems treating the LDF with 100 μg/ L of oxytetracycline, sulfadiazine and ofloxacin, respectively. The antibiotic concentration selected is an average of concentrations found in LFD (Yang et al., 2022). C, OX, SD and OF systems were set up all in triplicates, distributed randomly within the greenhouse, and were always treating 1 L of one of the four LFD types.

The LFD was recirculated over 14 days, then removed from the systems and replaced with fresh LFD, simulating the cumulative effect of full-scale CWs with a hydraulic retention time (HRT) of 14 days. In total, six 14-day cycles were performed between 1 September and 25 November 2021.

### 2.3 Samples collection and preservation

Samples of influent and effluent per CW, treatment and cycle were collected. Influent was sampled just before pouring it in the CWs for analysis of different parameters namely, pH, organic matter (estimated through COD), nutrients (ammonium, phosphate, nitrate, and nitrite ions), and metals (Fe, Mn, Zn, Ni, Cr, Cu, Pb). After each two-week treatment cycle, all the effluents from each CWs were collected in dark glass flasks, to protect them from light. After homogenization, different aliquots were collected to analyze the different parameters.

Influent samples for metals analysis were stored at −20°C, whereas fresh CWs effluent was acidified with 1% (v/v) nitric acid after collection and kept at 4°C until direct analysis.

Samples for pH and COD were collected and immediately analyzed. To analyze nutrients, aliquots of freshly collected influent and effluent samples were filtered through nitrate cellulose filters (0.45 mm) and kept at −20°C until analysis.

For the analysis of the antibiotic compounds, CWs effluents were filtered through glass fiber filters and concentrated by solid phase extraction (SPE) with Oasis HBL 3 cc (60 mg) Extraction Cartridges (Waters Corporation, Milford, MA, United States) immediately after sample collection using a vacuum manifold system (Supelco, Spain) coupled with a vacuum pump. SPE cartridges were eluted with a 96/4 (v/v) methanol/formic acid solution, adapting the methodology optimized by Cavenati et al. (2012). SPE extracts were kept at −20°C until analysis.

For the microbial community characterization and qPCR analysis, fresh CWs effluent samples of only the second, the fourth and the sixth treatment cycles were immediately filtered through Sterivex™ filter units with a pore size of 0.22 mm (Merck Millipore, Portugal), in duplicate, for 3 h until the filters were clogged. These sampling times were chosen to evaluate the monthly evolution of the communities. The filtered volume was on average 30 mL per sample (ranging from 7 mL to 42 mL). The inlet and outlet of these filter units were covered with parafilm, after removing the remaining liquid, and the Sterivex™ were kept in sterile plastic bags at −80°C. The initial LFD (influent) of the second, fourth and sixth cycles was also stored at −80°C right after the sampling.

### 2.4 Physicochemical analysis

pH was measured with a Crison micro pH 2002 with a Crison pH electrode in freshly collected samples. The COD content was determined using kits HI93754B-25 MR for a range 0–1,500 mg O_2_/L and HI93754A-25 LR for a range 0–150 mg O_2_/L and the absorbance of the samples was read in a HI83214 Multiparameter Bench Photometer (Hanna Instruments, Portugal). The concentration of ammonium, nitrite and phosphate ions was analyzed following the protocol described by Dias et al. (2020). The limit of detection (LOD) of ammonium, phosphate, nitrite, and nitrate ions was 0.05, 0.05, 0.01 and 0.1 mg/L, respectively.

For metal determinations, samples (2.8 g per sample) were digested in a high-pressure microwave system (Ethos, Millestone, Sorisole, Italy) with 1 mL of nitric acid and 5 mL of 30% of a hydrogen peroxide solution in microwave Teflon vessels. The microwave digestion program was: 5 min at 250 W, 5 min at 400 W, 5 min at 500 W and 10 min at 0 W, following a previously optimized lab protocol (Almeida et al., 2017b). Then, concentration of metals in microwave extracts and in the acidified CWs effluents were analyzed by atomic absorption spectrophotometry with flame atomization (Analyst 200AA spectrometer, PerkinElmer Inc., Waltham, MA, United States) for Fe, Mn, Zn, and Cu, and with electrothermal atomization (Atomic Absorption Spectrometer PinAAcle 900Z with Furnace Autosampler AS900, PerkinElmer Inc., Waltham, MA, United States) for Ni, Cr, and Pb, using external calibrations prepared with aqueous standard solutions for metal quantification. The LOD of Fe, Mn, and Cu were 0.1 mg/L, the one of Zn was 0.025 mg/L, whereas for Ni and Pb, LOD were 5 μg/L, and for Cr, 10 μg/L.

Before organic contaminant analysis, SPE extracts of CW effluents were evaporated until dryness and re-suspended in 200 μL of a 70/30 (v/v) methanol/water solution. The concentrations of oxytetracycline, sulfadiazine and ofloxacin antibiotics were analyzed by high-performance liquid chromatography (HPLC; Beckman Coulter Inc., Brea, CA, United States), adapting a previously optimized laboratory procedure (Cavenati et al., 2012). This equipment was coupled with a diode array detector (module 128) set up at 298 nm and an automatic sampler (module 508) and the antibiotics were separated in a 150 × 4.6 mm C18 Luna column (Phenomenex, United Kingdom). The LOD for oxytetracycline, sulfadiazine and ofloxacin were 0.8 μg/L.

### 2.5 Microbial community analysis

Microbial communities from CW influent and effluent samples were characterized using a 16S rRNA-based approach.

For the CWs effluents, liquid samples were collected, and DNA was extracted from Sterivex™ filters with the DNeasy PowerWater Sterivex Kit (QIAGEN Inc., Venlo, Netherlands). In the case of CWs influents, DNA was extracted from 0.5 g of LFD with the DNeasy PowerSoil Pro Kit (QIAGEN Inc., Venlo, Netherlands) following the manufacturer’s instructions and treating the samples as solid ones. DNA concentration and purity were determined by spectrophotometric analysis (NanoDrop ND-2000 and Qubit 4 Fluorometer, Invitrogen, MA, United States).

The prokaryotic community of influent and effluent samples along the different CWs treatment cycles was characterized by sequencing the V4 region of the *16S rRNA* gene targeting for both bacteria and archaea communities. For that, the V4 region of the *16S rRNA* gene was amplified using the primer pair 515FB (GTGYCAGCMGCCGCGGTAA) and 806RB (GGACTACNVGGGTWTCTAAT; Walters et al., 2016), according to the Earth Microbiome Project protocols. Sequencing of the amplicons was carried out on an Illumina MiSeq sequencer with the V3 chemistry (Illumina, San Diego, CA, United States) in Genoinseq, Biocant—Biotechnology Park (Cantanhede, Portugal).

The raw reads were pre-processed with PRINSEQ-Lite v0.20.4 that excluded reads shorter than 100 bp and an average quality lower than Q25 in a 5 bp window. The residual adapter sequences were removed with AdapterRemoval v 2.1.5. Then, all sequences were processed using R Software (v 4.1.2; R Core Team, 2021) in the DADA2 pipeline v 1.20.0 to filter, clean, dereplicate the sequences, infer amplicon sequence variants (ASVs) on forward and reverse reads, merge pair-end reads, and remove chimeras. The taxonomic assignment of the ASVs was performed with Silva v138 database using the Naïve Bayes classifier method (Quast et al., 2013; Yilmaz et al., 2014).

### 2.6 Quantification of ARGs

The abundance of five target genes (*intI1, tetA, tetW, sul1* and *qnrS*) encoding class 1 integron-integrase and resistance to oxytetracycline, sulfadiazine and ofloxacin were quantified through real-time qPCR. pGEM Easy with tetracycline-resistant genes and pNORM1 containing the other target genes were extracted from *Escherichia coli* strain CM865 and *E. coli* JM109, respectively with the QIAprep Spin Miniprep Kit (QIAGEN Inc., Venlo, Netherlands). The concentration of the purified plasmid was quantified by Qubit 4 Fluorometer (Invitrogen, MA, United States).

Plasmids were digested with FastDigest BamHI (Thermo Scientific, MA, United States) for 15 min at 37°C and the linearized products were purified with the PCR purification kit (QIAGEN Inc., Venlo, Netherlands). The eluted DNA was quantified with Qubit and these products were used to do serial dilutions of the target genes from 10^8^ to 10^1^ number of copies/μL standard curves to generate the standard curves, being 10^1^ copies/μL the LOD. 1 mL aliquots of standards were prepared with Equation 1.


(1) $$\frac{n^{{}^{\circ}}\;\mathrm{of}\;\mathrm{copies}}{\mathrm{\mu L}}=\frac{\begin{array}{l} \mathrm{concentration}\;\mathrm{of}\;DNA\;\left(\frac{g}{\mathrm{\mu L}}\right)\times \\ \mathrm{Avogadro}\;\mathrm{constant}\;\left(\frac{n^{{}^{\circ}}\;\mathrm{of}\;\mathrm{copies}}{\mathrm{mol}}\right) \end{array}}{\begin{array}{l} \mathrm{amplicon}\;\mathrm{size}\times \mathrm{molecular}\;\mathrm{weigh}\;\\ \mathrm{of}\;1\;\mathrm{bp}\;\mathrm{in}\;\mathrm{dsDNA}\;\left(\frac{g}{\mathrm{mol}}\right) \end{array}}$$

The qPCR analysis was performed in 96-well plates. Primer sequences, amplicon size and qPCR conditions of the different target genes are shown in Table 1. Each reaction was run in triplicate for DNA samples from the influent and effluents of CWs on a LightCycler 480 II platform (F. Hoffmann-La Roche AG, Basel, Switzerland). The reaction volume was 20 μL and consisted of 10 μL of LightCycler 480 Sybr Green I Master (F. Hoffmann-La Roche AG, Basel, Switzerland), 0.5 μL of both primers at 10 μM, 1 μL DNA template standardized at 20 ng/μL and 8 μL of nuclease-free water. The qPCR reactions were as described in Supplementary Table S1 followed by the melting curve step with temperature ramping from 60°C to 95°C to confirm the specificity of the amplicon.

**Table 1:** Average physicochemical parameters (*n* = 6) of the liquid fraction of digestate (during the six 14-day cycles) before treatment.

Parameter	Value
Total solids (TS)	9.6 ± 0.4%
Volatile solids	51 ± 1% TS
Inert particles < 0.5 mm	20 ± 4% TS
Density	0.95 ± 0.04 g/cm^3^
Temperature	23 ± 3°C
pH	8.15 ± 0.06
Conductivity	34 ± 1 mS/cm
COD	73 ± 4 g O_2_/L

To normalize the data, absolute abundances were represented by the number of gene copies within 1 mL of effluent or 1 g of influent samples. The relative abundances of ARGs were also calculated by dividing the number of copies of the target gene by the number of copies of 16S rRNA.

### 2.7 Data analysis and statistics

Each condition in CWs was tested independently in three microcosm systems in the same greenhouse and all the chemical analyses were also performed in triplicates. Means and standard deviations were calculated.

A Shapiro–Wilk test with *p* > 0.05 was carried out to confirm the normality of the dataset. A two-way analysis of variance (ANOVA) was used. Alternatively, Kruskal-Wallis one-way anova on ranks test was performed when the normality test was violated. A multiple comparison Tukey test was run to determine differences that were statistically significant between treatments and 14-days CWs treatment cycles for a 95% of confidence level with Sigmaplot software v 14.0. The removal efficiencies of the pollutants were calculated according to Equation 2.


(2) $$\mathrm{Removal}\;\mathrm{efficiency}\;\left(\%\right)=\frac{C_{\mathrm{in}}-C_{\mathrm{out}}}{C_{\mathrm{in}}}\times 100$$

where C_in_ and C_out_ are the concentrations of the target pollutant entering and leaving the different systems, respectively. Whenever the compound was not detected in the CWs effluent, the removal efficiency was calculated considering the value of the LOD of the respective analytical methodology for C_out_.

All bioinformatic analysis were performed with R software (v 4.1.2; R Core Team, 2021) and plotted with MicrobiomeAnalyst 2.0 (Lu et al., 2023). The number of raw reads from influent and effluent DNA samples ranged between 49,400 and 137,947 reads and after processing through the DADA2 pipeline the number of sequences decreased to between 34,920 and 97,368 (Supplementary Table S2). The alpha and beta diversity analysis were run with phyloseq package v 1.38.0 rarefying the number of ASV to 34,920 reads (the lowest number). On the one hand, the alpha diversity indexes analyzed were the observed ASVs, Shannon and Simpson indices at a featured level. On the other hand, beta diversity was studied with a non-metric multidimensional scaling using the Bray-Curtis index on the rarefied data followed by total sum scaling and removal of singletons. Dissimilarities between the ASVs distribution were examined with a permutational multivariate analysis of variance (permanova) with 999 permutations and with an analysis of similarity (anosim) with the vegan package v 2.5.2. Besides, the taxonomic composition of the microbial communities was performed at a phylum and genus level, also with the phyloseq package v 1.38.0. Genera associated with potential pathogenic bacteria were listed according to the 10 bacterial genera housing most pathogen species list (Bartlett et al., 2022), and then, its relative abundance in the CWs influent and effluent was calculated.

## 3 Results and discussion

### 3.1 Initial characterization

The six LFDs collected in the biogas plant to be treated in CWs exhibited very stable physicochemical characteristics despite being collected over a four-month period with seasonal shifts and operational adjustments of the biogas plant (Table 1). These effluents presented an average total solids content of 9.6%, density of 0.95 g/cm^3^, pH of 8.15, electrical conductivity of 34 mS/cm, and COD of 73 g/L, values consistent with previous reports (Akhiar et al., 2017). The concentrations of metals and nutrients in these LFDs are presented in Table 2. Ammonium, nitrate, nitrite, and phosphate ions amount in LFDs were, on average, 2,220, 89, 2.7 mg N/L, and 156 mg P/L, respectively. Moreover, iron levels exceeded the recommended concentration for irrigation water by more than 150 times, while the concentration of zinc, copper, manganese, and chromium was over 20 times the limits in the guidelines (World Health Organisation, 2006). Consequently, the collected LFD did not meet the minimum quality standards of the Water Reuse Regulation (European Commission, 2020), and exceeded the irrigation guidelines set by WHO for all the measured metals, except for lead (World Health Organisation, 2006), and by APA for total nitrogen and phosphorus (Ministério da Economia, 2015). Hence, proper treatment of LFD was needed to meet all these standards.

**Table 2:** Average concentration (*n* = 18) and standard deviation of metals and nutrients measured in the six initial liquid fractions of digestate (LFD; analysis in triplicate) and water quality standards guidelines of World Health Organisation (WHO) and Environmental Portuguese Agency (APA) for recommended metal and nutrient levels in irrigation with reused water (World Health Organisation, 2006; Ministério da Economia, 2015).

Metal	Concentration in LFD (mg/L)	WHO metal level (mg/L)
Fe	769 ± 93	5.0
Zn	40 ± 3	2.0
Cu	11 ± 1	0.2
Mn	7.4 ± 0.8	0.2
Pb	4.6 ± 0.9	5.0
Cr	2.7 ± 0.7	0.1
Ni	2.1 ± 0.3	0.2
**Nutrient**	**Concentration in LFD (mg N/L or mg P/L)**	**APA nutrient level (mg N/L or mg P/L)**
NH_4_^+^-N	2,220 ± 119	10
NO_3_^−^-N	89 ± 17	TN = 15
NO_2_^−^-N	2.7 ± 1.5	TN = 15
PO_4_^3−^-P	157 ± 36	TP = 5

### 3.2 CWs treatment efficiency

#### 3.2.1 Physicochemical parameters

**Figure 1:**
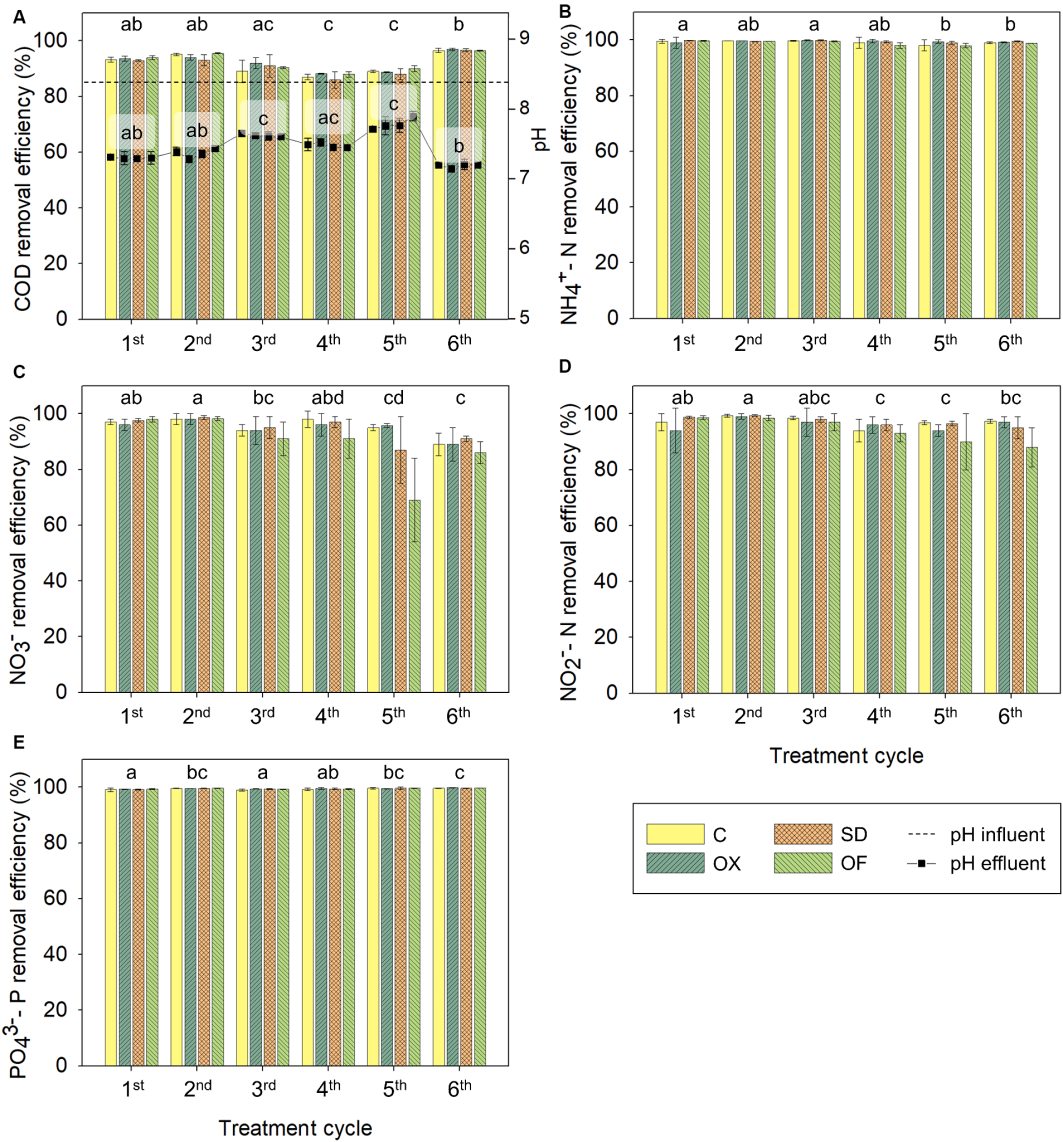
Average removal percentages of organic matter **(A)**, ammonium **(B)**, nitrate **(C)**, nitrite **(D)**, and phosphate **(E)** ions in CWs treating in parallel the 4 different LFD during six 14-days treatment cycles. In panel **(A)**, the dashed line indicates the average pH of the six influents and the dots represent the average pH of the effluents for each treatment. There were no significant differences in pH, COD or nutrient concentration between the different LFD except in the 5th cycle where OF treatment presented significant differences with both C and OX treatment. Only statistical data regarding the treatment cycles is presented and the same letters indicate that subsets are not significantly different at *p* < 0.05 by ANOVA on ranks. COD, chemical oxygen demand; LFD, liquid fraction of digestate; C, control LFD; OX, LFD doped with oxytetracycline; SD, LFD doped with sulfadiazine; OF, LFD with ofloxacin.

The pH variation and the COD removal percentage are presented in Figure 1A. On the one hand, during CWs treatment, pH decreased from above 8 to a range between 7.1 (in the sixth cycle) and 8.0 (in the fifth cycle), with significant variations among the effluents of different cycles. No significant differences were observed within each cycle among treatments, except in the fifth cycle. On the other hand, CWs microcosms showed effective COD removal, ranging from 82 to 98%, always below 2 g O_2_/L in CWs effluent (Supplementary Table S3). Although no significant differences were found among treatments within any cycle, significant variations were observed between cycles. These removal rates were in line with other studies of two hybrid pilot CWs treating digestate from a digester fed with livestock waste, where the percentages of reduction of COD were 88% and 89.2% (Comino et al., 2013; Maucieri et al., 2016). However, other studies treating LFD with CWs presented lower removal rates of COD, between 52% and 73% (Wu et al., 2016b; Zhou et al., 2020). The higher removal rates observed in the present case could be attributed to the low organic loading and flow rate, specifically 8.87 g COD/m^3^·d and 2.57 L/d, respectively. In vertical subsurface flow CWs, the removal of organic matter is caused by physical, chemical, and biological processes. Physical processes such as filtration and sedimentation, occurring in the substrate layers, are primarily responsible for the retention of particulate organic matter that is hydrolyzed into humic-like substances, generating soluble organic matter. This soluble organic matter is oxidized and degraded by aerobic microbial metabolism (García et al., 2010).

Furthermore, the concentration of ammonium and phosphate ions in CWs effluents were below 10 mg N/L in cycles 1, 2, 3, and 6, and below 1.0 mg P/L in all cycles and treatments, respectively (Supplementary Table S4). Results of nutrient removal from the LFD are shown in Figures 1B–E. CWs removed over 98% of ammonium and phosphate, 69% of nitrate, and 90% of nitrite ions, with no significant differences among treatments, except in the fifth cycle where significant differences were observed between both C and OX, with OF treatment. Similar results have been obtained in other studies treating LFD in CWs (Comino et al., 2013; Nakamura et al., 2017), and in studies, previously published by the authors, treating wastewater spiked with antibiotics (Almeida et al., 2017b; Santos et al., 2019), indicating consistent nutrient removal across different experimental conditions, independently of the presence-absence of tested antibiotics in the influent.

The mechanisms involved in nutrient removal encompass a combination of mechanical and biogeochemical processes, including sedimentation, adsorption, volatilization, chemical precipitation, nitrification–denitrification, plant, and microbial uptake, and rhizofiltration (Kamilya et al., 2022). On the one hand, phosphate ions are removed primarily through sorption, sedimentation, and plant uptake (Vymazal, 2007). On the other hand, nitrogen removal is mainly driven by microorganisms through ammonification, nitrification, and denitrification (Wang J. et al., 2022). Vertical subsurface flow CWs, characterized by higher oxygen capacity, exhibit enhanced removal of ammonium ions compared to horizontal subsurface flow CWs (Vymazal, 2007; Kamilya et al., 2022). In the present study, despite the daily recirculation to promote aerobic conditions, there are still some anoxic zones in the systems that could favor conditions for facultative anaerobes to facilitate denitrification processes, thus explaining the variability in nitrate and nitrite concentrations observed among systems. However, from the third cycle onwards, there was a tendency for a slight decrease on nitrate and nitrite removal for all the systems, being the removal rates of nitrates in OF systems the lowest. Previous studies have reported that 10 μg/L of ofloxacin reduced the nitrate removal efficiency from 83.40% to 40.20%, due to the inhibition of denitrifying gene expression and denitrifying bacteria activity (Tong et al., 2019; Zhang et al., 2022).

**Figure 2:**
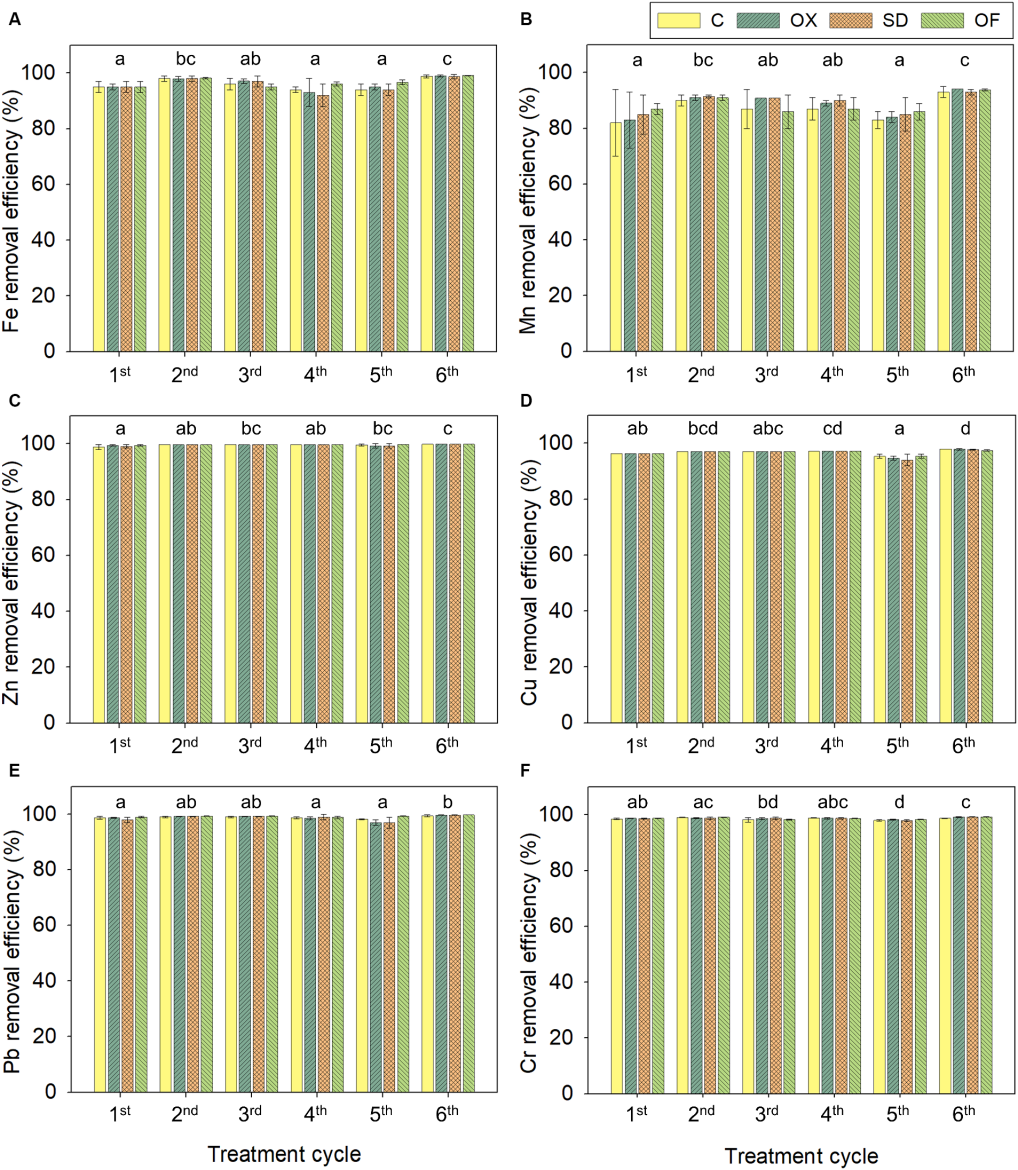
Average removal percentages of metals in CWs treating in parallel the 4 different LFDs during six 14-days treatment cycles: iron **(A)**, manganese **(B)**, zinc **(C)**, copper **(D)**, lead **(E)**, and chromium **(F)**. The same letters indicate that the cycles subsets are not significantly different at *p* < 0.05 by ANOVA on ranks (there were no signigicant differentces between LFD treatments). LFD, liquid fraction of digestate; C, control LFD; OX, LFD doped with oxytetracycline; SD, LFD doped with sulfadiazine; OF, LFD with ofloxacin.

In addition, in this study, CWs showed also high removal efficiency of metals (Figure 2). Over 94% of zinc, copper, lead, and chromium were removed in all cycles from all LFD after CWs treatment. Regarding the removal of iron and manganese, the second and sixth cycles exhibited the highest rates, over 98% and 90%, respectively, while in the other cycles, the removals were slightly lower ranging between 92% and 97% for iron, and between 82% and 91% for manganese. No significant differences among treatments were observed, except for copper and lead, where its removals in the fifth cycle presented differences. Overall, the concentrations of manganese, zinc, copper, lead, and chromium in the effluent were in all cases below the concentrations of the WHO guidelines for irrigation water, the highest concentrations being 0.2, 0.07, 0.09, 0.006, and 0.009 mg/L, respectively (Supplementary Table S5). However, the concentration of iron exceeded the recommended limits in the third, fourth and fifth cycles reaching concentrations up to 9 mg/L. High removals of metals in CWs have been published in systems treating pig industry effluents, with removals over 85% for iron, zinc and copper and slightly lower removals of manganese too (Almeida et al., 2017a). The main processes contributing to the removal of metals from effluents are sedimentation, filtration, adsorption (co-)precipitation, plant ad/absorption and microbial immobilization (García et al., 2010; Yu et al., 2022).

#### 3.2.2 Antibiotics

In CWs effluents, the concentration of oxytetracycline, sulfadiazine and ofloxacin was below the LOD in all cycles and treatments, indicating a removal efficiency above 99%. These high antibiotic removals are in accordance with previous studies evaluating the performance of CWs removing veterinary antibiotics from livestock wastewater (Carvalho et al., 2013; Almeida et al., 2017a; Santos et al., 2019). These high removals can be attributed to several factors: the vertical subsurface flow configuration facilitating the chemical oxidation and the growth of aerobic communities; a high HRT promoting biodegradation of antibiotics by microbial communities; the combination of different substrates (sand, LECA and gravel) promoting filtration and adsorption of molecules with different chemical properties. Also, the presence of *S. erectum* might enhance plant uptake, microbial growth, and adsorption too (He et al., 2021; Lv et al., 2022). However, biodegradation can be the principal removal mechanism of oxytetracycline, sulfadiazine and ofloxacin, driven by various microorganisms and different metabolic pathways (Ma et al., 2022). Other complementary removal pathways could be *S. erectum* uptake by water transport and passive absorption for oxytetracycline (because of its low octanol–water partition coefficient), methylation and oxidation for sulfadiazine, and formation of complexes with dissolved organic matter for ofloxacin, further degraded by the carbon metabolism and denitrifiers (Lv et al., 2022; Liu et al., 2023).

Although antibiotics can alter microbial communities and have toxic effects to both microorganisms and plants in CWs (Ohore et al., 2022), that was not the case for the present study. At the tested antibiotic concentration and CWs conditions, there were no significant differences in the removal of COD, ammonium and phosphate ions and metals. These findings indicate that the presence of antibiotics did not negatively affect the performance of CWs systems. The fact that there were generally no discernible changes in the concentrations of the many parameters examined over time suggests that the system remained functional throughout the 3 months of the experiments. Following the removal kinetics proposed by Dan et al. (2021), the amount of antibiotics could have been rapidly adsorbed and removed from the effluent (in 1 day approximatively), explaining the absence of significant differences between treatments.

### 3.3 Prokaryotic community diversity and composition

#### 3.3.1 Diversity analysis

**Figure 3:**
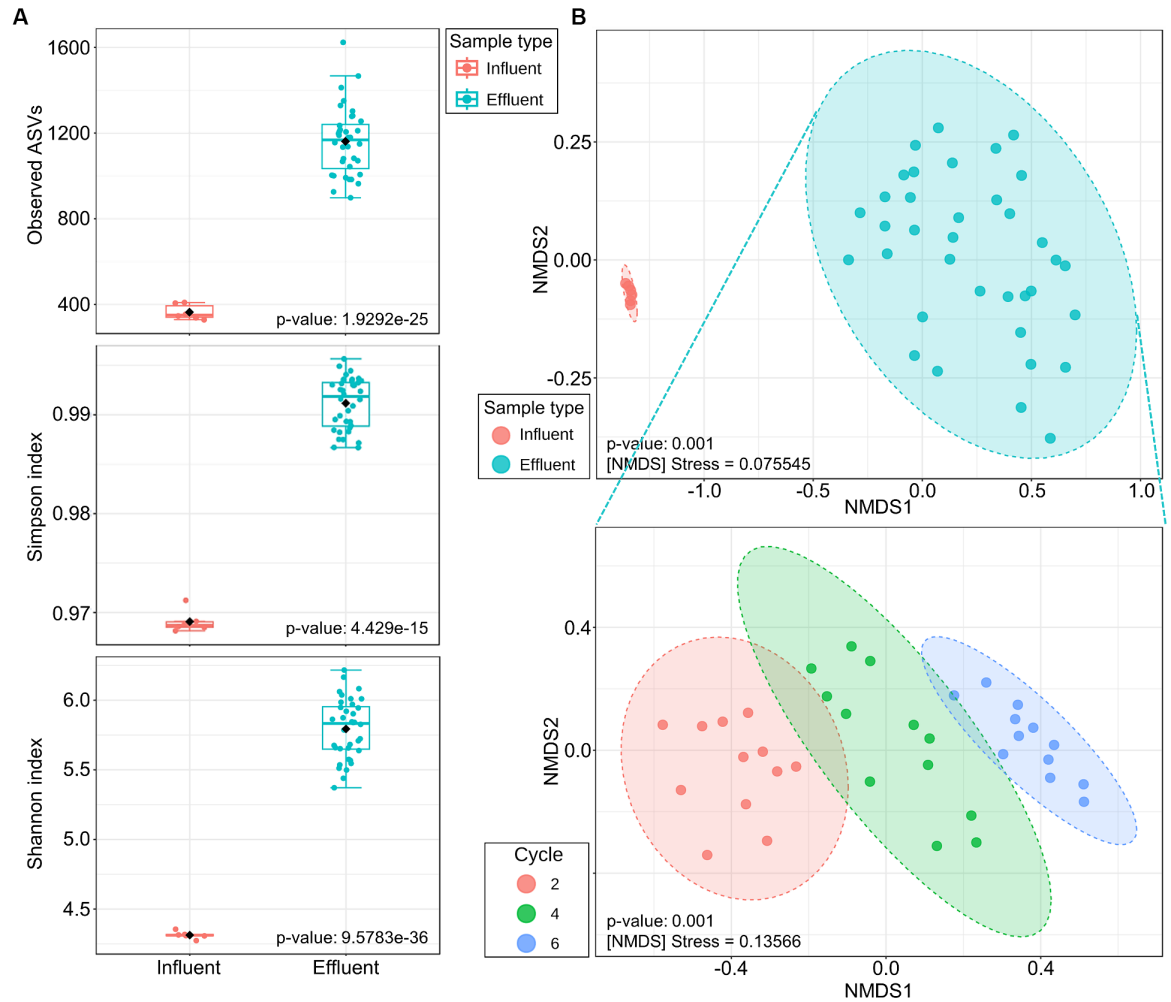
**(A)** Box plots of three alpha diversity indexes, observed ASVs, Simpson index, and Shannon index, in the LFD before and after the 14-days treatment in CWs of the second, fourth and sixth CWs treatment cycles. The bottom, center, and top of each box correspond to the 25^th^, 50^th^, and 75^th^ percentiles, respectively and error bars show the 95% confidence range. **(B)** Non-metric multidimensional scaling plots with Bray-Curtis index of all the CWs samples, including both influent and effluents samples (on the top), and of the effluent samples of the 2^nd^, 4^th^, and 6^th^ cycle (on the bottom). CWs influents of each cycle are in duplicates, and the effluent samples in triplicates per each treatment and per cycle.

The prokaryotic community diversity within CWs influent and effluent samples is summarized by analyzing the richness with the observed ASVs, and other diversity indexes, namely Simpson and Shannon indexes (Figure 3A). Around 400 different ASVs were detected in influent samples whereas 1,150 on average were observed in the effluent (Figure 3A). Thus, samples after the treatment in CWs exhibited higher richness in microbial community (almost 3 times more) than the LFD before the treatment.

However, both influent and effluent samples presented Simpson index values close to 1, indicating low species evenness, where few groups dominated the community. Figure 3A shows that communities in the effluent were less even than in the influent.

Regarding Shannon index, which considers both the richness and evenness of ASVs within a sample, there was a significant increase in diversity in the effluent compared with the digestate samples before treatment (Figure 3A).

The beta-diversity analysis of the LFD before and after the treatment in CWs was performed through a non-metric multidimensional scaling using the Bray-Curtis index. Figure 3B showed significant dissimilarities between the community structures of the CWs influent and the effluent samples, with no distinctions among the different treating LFD conditions (C, OX, SD and OF). The stress value of 0.076 confirmed the good representation in the plot of the distance matrix of influent and effluent data.

When focusing on the diversity between samples after the treatment in CWs, effluent samples of the same treatment cycle were clustered together. Significant differences were, however, observed among communities of the second, fourth and sixth CWs treatment cycles, showing distinctions in the communities along time (Figure 3B).

#### 3.3.2 Taxonomic composition

**Figure 4:**
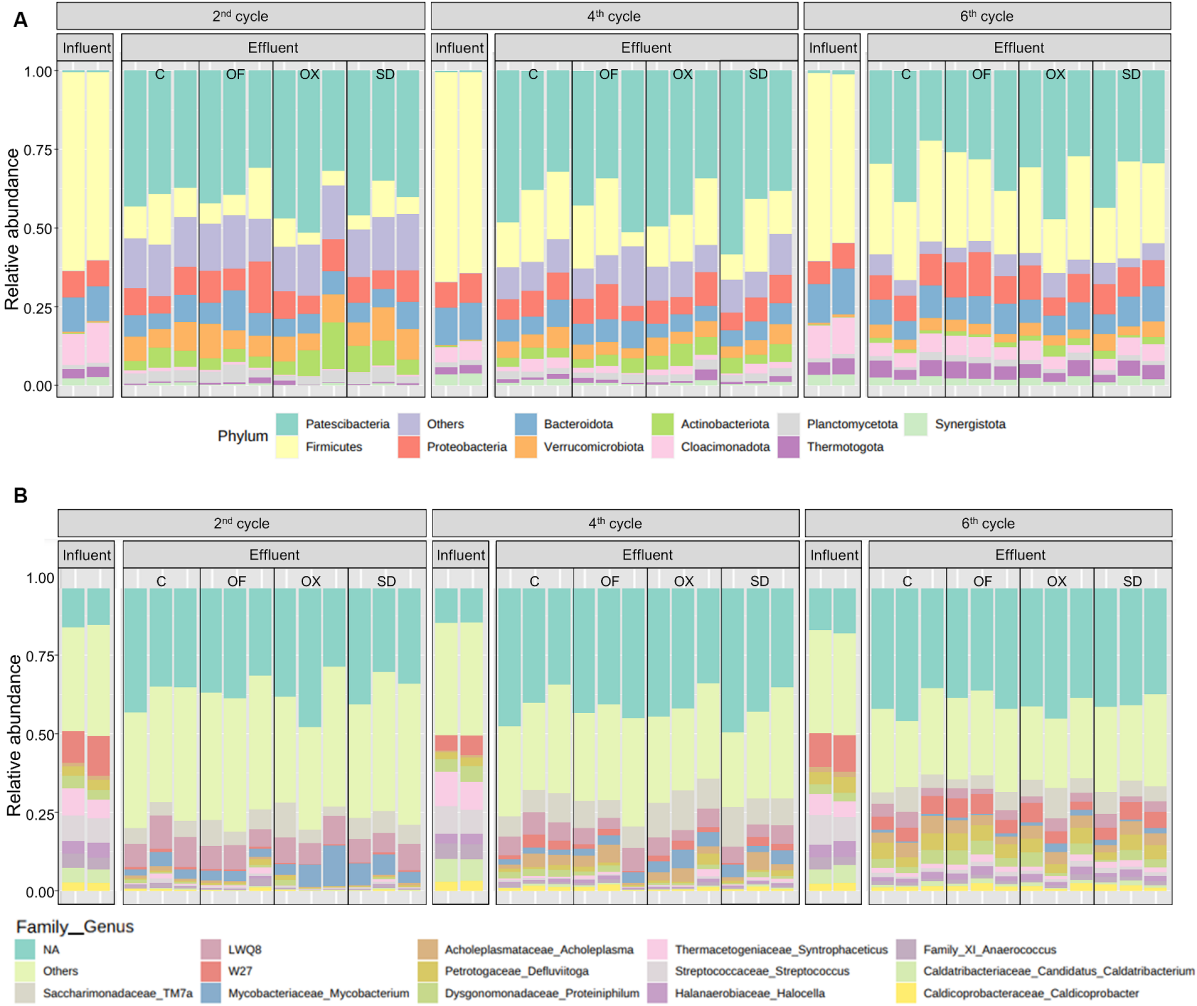
Taxonomic profile of prokaryotes in the influent and effluent of CWs based on the most relatively abundant at the phylum level (**A**, top 10 phyla), and at the family and genus level (**B**, top 14 genera). Each black box shows the replicates. C, LFD control; OX, LFD spiked with oxytetracycline; SD, LFD with sulfadiazine; OF, LFD with ofloxacin.

CWs influent, LDF, showed a consistent microbial community over time, with 16–18 phyla identified, while in CWs effluent 34 phyla were counted on average. Figure 4A presents the 10 prokaryotic phyla with the highest abundance, all belonging to the bacterial domain. Before CWs treatment, the LFDs population was dominated by Firmicutes, accounting for 53–66% of the community. This phylum is frequently detected in digestate and other livestock effluents (Koniuszewska et al., 2021; Bôto et al., 2023; Pan et al., 2023). Cloacimonadota and Bacteroidota, with relative abundances of 9 and 8% on average, respectively, were the following phyla more abundant, also reported previously in digestates (Koniuszewska et al., 2021; Blasco et al., 2022; Pan et al., 2023). On the one hand, Firmicutes and Bacteroidota are well-known for their ability to break down volatile fatty acids (VFA), and they can tolerate variations in temperature, pH, and oxygen levels. Additionally, they are characterized by their potential for hydrolysis and hydrogenogenic acidogenesis. Bacteroidota are not only involved in protein degradation but also produce lytic enzymes and acetic acid and are commonly found in anaerobic digestion processes using various substrates (Koniuszewska et al., 2021). On the other hand, Cloacimonadota bacteria, commonly found in engineered and wastewater systems, exhibit acetogenic and fermentative metabolism. They contribute to the carbon and energy cycling processes and are involved in the degradation of lipids and long-chain VFA (Johnson and Hug, 2022).

Different trends in relative abundances were noted between the CWs influent and effluent bacterial communities, with no discernible pattern between the different treatments (C, OX, SD, and OF) in each cycle (Figure 4A). After the treatment in CWs, Patescibacteria became the most abundant phylum (between 22% and 59%), and while no studies have been found, to the best of our knowledge, that specifically identify Patescibacteria as the dominant phylum in CWs effluents, its presence has been reported in the episphere of *Vallisneria natans* leaves in CWs treating water contaminated with erythromycin and in the rhizosphere of *Iris pseudacorus* in CWs treating wastewater spiked with enrofloxacin (Ramdat et al., 2022; Chen et al., 2023). Patescibacteria, formerly referred to as Candidate Phyla Radiation, represents a superphylum characterized by being obligate fermenters and playing important roles in subsurface carbon and nitrogen cycling. These microorganisms are commonly found in groundwater environments with a preference for oxic conditions and planktonic growth (Danczak et al., 2017; Gios et al., 2023). In this study, this abundant phylum was followed by Firmicutes, Proteobacteria, Bacteroidota, and Verrucomicrobiota. Proteobacteria, widely detected in CWs substrates and effluents, comprises the main functional microorganisms involved in the removal of organic matter, nitrogen, and antibiotics from various types of wastewaters (Wang J. et al., 2022; Bôto et al., 2023).

Along CWs treatment cycles, a diminishing trend was observed for Patescibacteria, Proteobacteria, Verrucomicrobiota and Actinobacteria in the effluents. On the contrary, Firmicutes’ relative abundance in samples of the last cycle (17%–33%) increased in comparison with the second one (3%–17%), and Bacteroidota, Cloacimonadota and Thermotogota showed a similar rising trend. The increase of Firmicutes and Bacteroidota in the effluent over time suggested a potential loss of CWs removal capacity of microorganisms from the influent or an enhancement of their growth conditions within the systems. These two phyla, commonly detected in CWs, are also related with the removal of pollutants (Wang J. et al., 2022).

At the genus level, prokaryotic communities of the CWs influent and effluent presented different taxonomic profiles (Figure 4B). In both influent and effluent samples, non-assigned taxa and less abundant ASVs (other than the 14 most abundant genera) were predominant in the community. *Streptococcus* and *Syntrophaceticus* were the genera in CWs influent with the highest abundance (around 8%). In addition, 6 additional bacterial genera presented a relatively even distribution in the LFD before treatment in CWs: *Candidatus Caldatribacterium* (5.7%), *Halocella* (4.2%), *Anaerococcus* (4.1%), *Proteiniphilum* (3.7%), *Defluviitoga* (3.3%), and *Fastidiosipila* (3.0%). These genera are commonly found in anaerobic digestors as carbohydrates degraders, sugar fermenters, proteolytic bacteria, or syntrophic acetate-oxidizing bacteria (Lim et al., 2020; Kostopoulou et al., 2023).

However, in the effluent there were fewer dominant genera, namely *Mycobacterium* (1.9%–12.9%) and *TM7a* (2.8%–10.9%) after the first month of the experiment, and *TM7a* (2.9%–9.6%), *Acholeplasma* (2.4%–5.5%), *Defluviitoga* (2.3%–5.4%), and *Proteiniphilum* (0.7%–4.7%) after the third month. *Mycobacterium* was found to be involved in denitrification and in the co-metabolism of organic matter and antibiotics in CWs, and another sulfonamide, sulfamethoxazole, promoted the growth of this bacteria such (Qu et al., 2022). In this study, the relative abundance of *TM7a* and non-assigned bacteria from LWQ8 family, both belonging to the Saccharimonadales order, also increased notoriously after the treatment in CWs. Previous studies have also reported the presence of *TM7a* in CWs effluents, ranging from 2.8% to 10.9% of the community (Cheng et al., 2022). Saccharimonadales, related to the carbon and nitrogen cycles, were proposed as potential bioindicators of elevated phosphorus levels and were found to be predominant in environments with high organic content. These bacteria exhibit synergistic interactions with genera associated to nitrification and denitrification (Wang G. et al., 2022). Hence, the effluent microbiome showed higher abundance of genera than the influent, resulting in a bacterial diversification that potentially contributed to higher pollutant removal, enhanced different metabolic pathways, and increased stability in the ecosystem, as previously observed (Choi et al., 2022; Bôto et al., 2023).

Moreover, numerous genera detected in the effluent are related to functional microorganisms with a crucial role in the removal of nitrogen (Saccharimonadales, *Candidatus Nitrotoga, Candidatus Omnitrophus, Denitratisoma*, *Gemmobacter*, *Thermomonas*), phosphorus (Rhodobacteraceae, Anaerolineaceae, *Dechloromonas, Acinetobacter*, and *Brevundimonas*), metals (*Desulfovibrio, Geobacter, Sideroxydans, Hydrogenophaga,* and *Chryseobacterium*) and antibiotics (Wang G. et al., 2022). Certain bacterial genera detected in effluents of OX systems namely Comamonadaceae bacteria, *Dechloromonas*, *Thiobacillus* and *Mycobacterium* were reported to be involved in oxytetracycline degradation. *Bacillus*, *Geobacter*, and unclassified Gemmatimonadaceae bacteria, detected in low abundances in SD systems effluents, were involved in sulfadiazine degradation. *Rhizobacter* and *Bacteroides*, detected in OF systems effluents, were associated with ofloxacin degradation (Chen et al., 2019, 2022; Wang J. et al., 2022).

### 3.4 Removal of potential pathogens and ARGs

**Figure 5:**
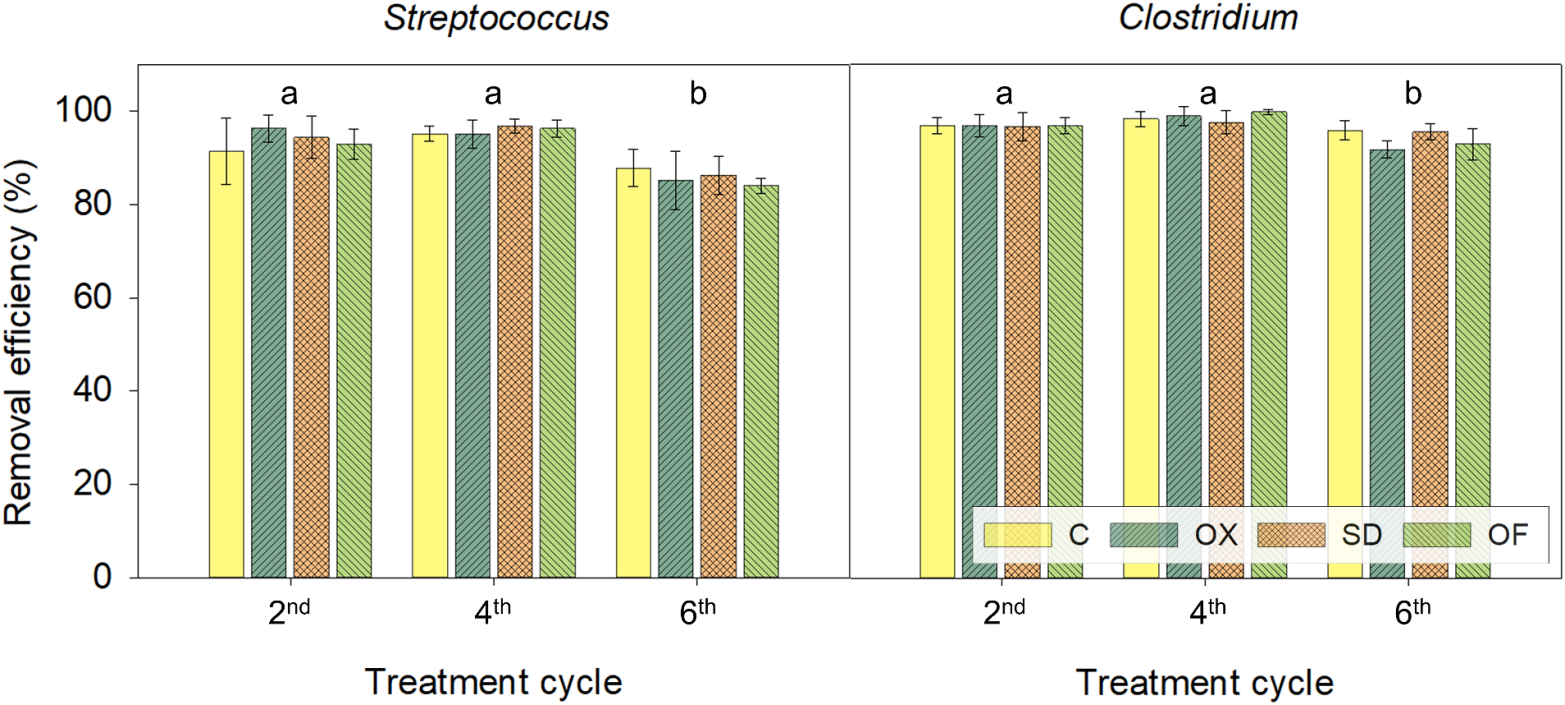
Average removal percentages of *Streptococcus* and *Clostridium* genera in CWs treating in parallel the 4 different LFDs during the second, the fourth and the sixth 14-day treatment cycles. The same letters indicate that the cycles subsets are not significantly different at *p* < 0.05 by two-way ANOVA (there were no significant differences between LFD treatments). C, control digestate; OX, digestate doped with oxytetracycline; SD, digestate doped with sulfadiazine; OF, digestate with ofloxacin.

Although anaerobic digestion (especially at thermophilic conditions) is effective in reducing most of the pathogens from livestock and other organic waste, residual pathogenic bacteria are still present in LFD posing a risk when reusing these effluents in agriculture. Pathogens commonly found in digestates are coliform bacteria, *Salmonella, Staphylococcus aureus, Mycobacterium paratuberculosis*, and Streptococci. *Streptococcus faecalis* is an indicator of the sanitation efficiency of digestates because is one of the most resilient organisms in anaerobic digestion processes compared to other hazardous bacteria, viruses, and parasites (Al Seadi et al., 2010). In this study, *Streptococcus* and *Clostridium* (*sensu stricto* 1, 8 and 15) were the potential pathogenic genera detected in LFD, the former in high abundance (Supplementary Table S6). The relative abundance of *Streptococcus* decreased with CWs treatment, with removal percentages averaging 94%, 96%, and 85% in the second, fourth, and sixth treatment cycles, respectively (Figure 5), suggesting a persistence of this potential pathogen ranging from 6% to 15%. A similar tendency was observed for *Clostridium* with removals of 97%, 99%, and 94% on average in these treatment cycles, suggesting a persistence of this potential pathogen ranging from 1% to 6%. Although associated with pathogenic bacteria, both genera are also involved with essential metabolic pathways in anaerobic digestion processes. *Streptococcus* can be strictly fermenters producing VFA, ethanol, H_2_ and CO_2_ and *Clostridium* can contribute to biomass breakdown, participate in acetogenesis, and produce various extracellular enzymes that degrade biopolymers, leading to improved methane production (Zhang et al., 2017; Yao et al., 2019).

**Figure 6:**
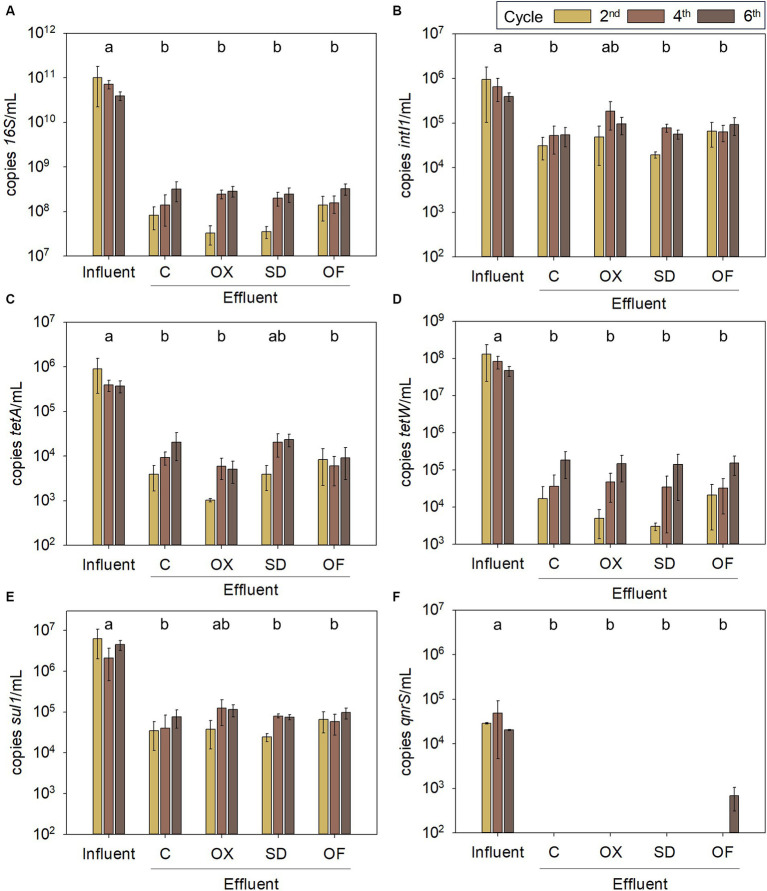
Absolute abundance of V3 region of *16S rRNA* **(A)**, *intI1* **(B)**, *tetA* **(C)***, tetW* **(D)***, sul1* **(E)**, and *qnrS* **(F)** genes in LFD before (influent) and after (effluent) the treatment in CWs. In the legend, numbers 2^nd^, 4^th^, and 6^th^ indicate the number of the two-week treatment cycle. The letters correspond to the subsets that are not significantly different at *p* < 0.05 by ANOVA on ranks. C, control digestate; OX, digestate spiked with oxytetracycline; SD, digestate with sulfadiazine; OF, digestate with ofloxacin.

Other contaminants with growing concern in wastewater treatment processes are ARGs. Despite the removal of ARGs and MGEs during anaerobic digestion, these genes can remain in the LFD (Gurmessa et al., 2020). The potential transfer of ARGs to bacterial pathogens poses a significant global public health issue. However, the present study reveals a notable reduction of ARGs absolute abundances (10 to 1,000 times) after LFD treatment in CWs, in line with the substantial decrease of the total bacteria marker (*16S RNA*), from 7 × 10^10^ in the influent to around 1.8 × 10^8^. This indicates a huge reduction of bacteria in the effluent (Figure 6). *intI1* genes concentration in the influent was 6.7 × 10^5^ copies/mL on average, which further decreased to 7.0 × 10^4^ copies/mL, on average, after the treatment. Also, *intI1* relative abundance among the bacterial community was almost negligible (Supplementary Figure S2), suggesting poor potential for MGEs transference. The measured ARGs were also in a very low abundance in the microbial community of the CWs influent. More specifically, the resistance genes to oxytetracycline (*tetA* and *tetW*) were notably reduced 100 and 1,000 times (from 5.6 × 10^5^ to 9.8 × 10^3^, and from 8.7 × 10^7^ to 6.8 × 10^4^ copies/mL), respectively, on average, in line with prior research (Huang et al., 2017). Nonetheless, the removal rates of these genes exhibited a decreasing trend across successive cycles. Additionally, *sul1* gene, encoding resistance to sulfadiazine, was reduced along CWs treatment from 4.3 × 10^6^ to 6.9 × 10^4^ copies/mL on average. Finally, *qnrS* gene abundance, encoding resistance to ofloxacin, was below the LOD in the influent and in the effluent of the second and fourth cycles. In the sixth cycle, only systems treating LFD spiked with ofloxacin showed a concentration of *qnrS* gene copies above the LOD, reaching 6.2 × 10^2^ copies/mL. The increase in *qnrS* was also observed in Sun and Zheng (2023).

ARGs could have been eliminated mainly through plant uptake, die-off of bacterial hosts or sorption to organic matter (Sabri et al., 2021). The low ARGs discharge in CWs potentially minimized the transference of ARGs to pathogens. However, previous studies have shown that ARGs relative abundance can increase in CWs treating wastewater with antibiotics (Ohore et al., 2022). In fact, this was also observed in the present study, as the relative abundance of *intI1, tetA* and *sul1* increased in CWs effluents compared to CWs influent with no significant differences between the presence or absence of antibiotics (Supplementary Figure S2), suggesting that the selective pressure of antibiotics was not the only mechanism promoting this increase. Metals and high HRT (considering 14 days as a high HRT) are two factors that could have induced ARGs proliferation (Ohore et al., 2022). An option to reduce the ARGs dissemination in the environment in the long term could be to combine the systems with advanced treatment technologies such as advanced oxidation processes or membrane filtration (Monsalves et al., 2022).

Nevertheless, the removal of pathogens and bacteria with ARGs in CWs is driven by a combination of many factors. Sedimentation has been shown to effectively remove *Streptococcus* and other bacteria with high settling velocity (Wu et al., 2016a). Moreover, these removals were related to high removals of COD removals of due to the attachment of bacteria in retained organic matter particles. Other processes that could have happened are mechanical filtration in sand, adsorption mainly in LECA and plant roots, and natural die-off because of inactivation processes such as predation and starvation (Wu et al., 2016a).

Overall, the high removal of all pollutants obtained could be attributed to the high HRT (14 days), as many studies reported that the residence time of treatment significantly impacts the removal of pollutants (Yuan et al., 2022). This study makes a valuable contribution to the application of vertical subsurface flow CWs with gravel, LECA and sand, planted with *S. erectum* as a decentralized wastewater treatment technology to treat LFD with metals and antibiotics for water reuse purposes.

Depending on the CWs’ load, systems are expected to last over two decades, up to 20 years or more (Dotro et al., 2017). Previous long-term research showed that despite initial high concentrations of metals in the influent, CWs maintained high removal efficiencies, with metals often deposited in sediments in a non-bioavailable form (Knox et al., 2021) or accumulated by plants. Similarly, long-term studies reported high nutrient removal percentages in CWs (Nilsson et al., 2020). When the vegetation management techniques are appropriate, like seasonally harvesting, and eventually removal of accumulated solids, the removal efficiencies of CWs can be maintained over the years (Vymazal, 2020). Regarding scalability, although some previous pilot-plant studies showed similar removals as those achieved in lab-scale previous works, when scaling up the CW prototypes, deviations in efficiencies could be observed (Saúco et al., 2021). Hence, future research should focus on evaluating the potential of CWs to remove metals, antibiotics and ARGs from LFD at a pilot scale in the long term, optimizing the operational parameters, adapted to the digestate volume produced annually by the biogas plant. Additionally, further studies testing the dimensions, the systems’ shape and the hydraulic characteristics and configuration are necessary to prevent potential clogging problems or the dissemination of pollutants.

## 4 Conclusion

The present work studied the performance of CWs to treat the liquid effluents of anaerobic digesters, to allow its reuse in irrigation, taking into account both chemical and biological contaminants, including potential pathogens and ARGs. The results showed that CWs removed COD, ammonium, nitrates, nitrites, and phosphate ions at rates over 86%, 98%, 69%, 90%, and 98%, respectively. The systems reduced the metal levels between 88.2% and 99.5% for Fe, between 68.8% and 94.0% for Mn, over 97.8% for Zn, over 92.4% for Cu, over 95.9% for Pb and over 97.3% for Cr, with no significant differences between the four treatments (LFD spiked with oxytetracycline, with sulfadiazine, or with ofloxacin or without dosing).

After the treatment in CWs, concentrations of oxytetracycline, sulfadiazine and ofloxacin were below the detection limit in all systems, indicating successful removal. For most of the ARG analyzed (*intl1*, *tetA*, *tetW* and *sul1*), the absolute abundance decreased after the treatment of LFD in CWs. However, a slight increase in the relative abundance of some these ARG (*intl1*, *tetA*, and *sul1*) was observed, with a tendency to diminish over time.

Moreover, prokaryotic communities presented higher diversity after the treatment in CWs with significant differences between the community structures of the CWs influent and the effluent samples. Although no significant changes in the community were detected between treatments (presence or absence of antibiotics in the influent), there was a clear differentiation in the effluent’s communities over time. Removal of the potential pathogenic genera were observed, above 85% for *Streptococcus* and 94% for *Clostridium.* Overall, CWs are a suitable alternative to valorise the liquid effluents of anaerobic digesters, allowing its reuse in irrigation, closing the loop under a circular bioeconomy model, contributing to sustainable development goals of the 2030 Agenda (SDG6, SDG7, SDG11). However, the dissemination of ARGs in the environment remains a grand challenge that needs further understanding and management for their proper removal, and wastewater treatment solutions must consider this aspect to mitigate potential risks.

## Data availability statement

The sequencing data is available in the Sequence Read Archive of National Center for Biotechnology Information with accession number PRJNA1089588.

## Author contributions

PP-S: Conceptualization, Data curation, Formal analysis, Investigation, Methodology, Validation, Visualization, Writing – original draft. MT: Resources, Writing – review & editing. JF: Methodology, Resources, Writing – review & editing. AMe: Supervision, Validation, Writing – review & editing. BF: Supervision, Writing – review & editing. GC: Conceptualization, Funding acquisition, Project administration, Writing – review & editing. MA: Resources, Writing – review & editing. RC: Resources, Writing – review & editing. CG: Resources, Supervision, Writing – review & editing. CA: Conceptualization, Funding acquisition, Methodology, Project administration, Resources, Supervision, Validation, Writing – review & editing. AMu: Conceptualization, Funding acquisition, Methodology, Project administration, Resources, Supervision, Validation, Writing – review & editing.
