# Supplementary material for: Removal of metals and emergent contaminants from liquid digestates in constructed wetlands for agricultural reuse
Source: Front Microbiol. 2024 Jun 6;15:1388895. doi: 10.3389/fmicb.2024.1388895 (PMC11187104; doi:10.3389/fmicb.2024.1388895)
Supplement: Supplementary file 1 [file Data_Sheet_1.DOCX]

Supplementary Material


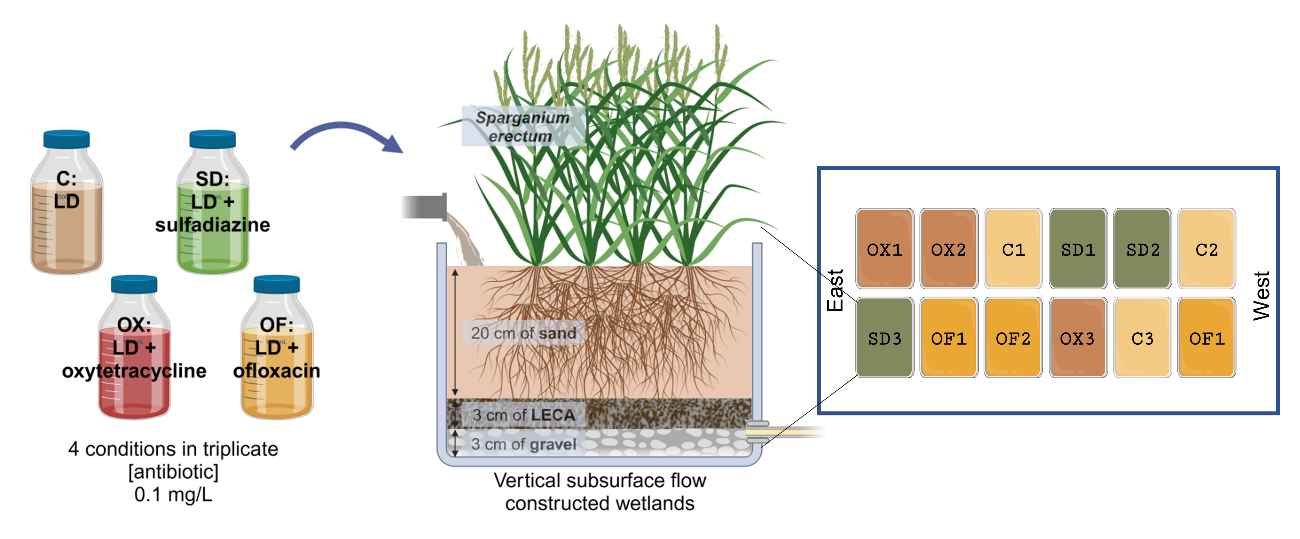


**Supplementary Figure 1.** Diagram of vertical subsurface flow CWs microcosms experiments. At the left, the four liquid digestate (LD) to be treated in the systems (with or without antibiotics), in the middle the different layers of substrate and the CWs’ vegetation and in the right the different system distribution in the greenhouse. Created with BioRender with the publication licence nº SE267I8KKZ.

**
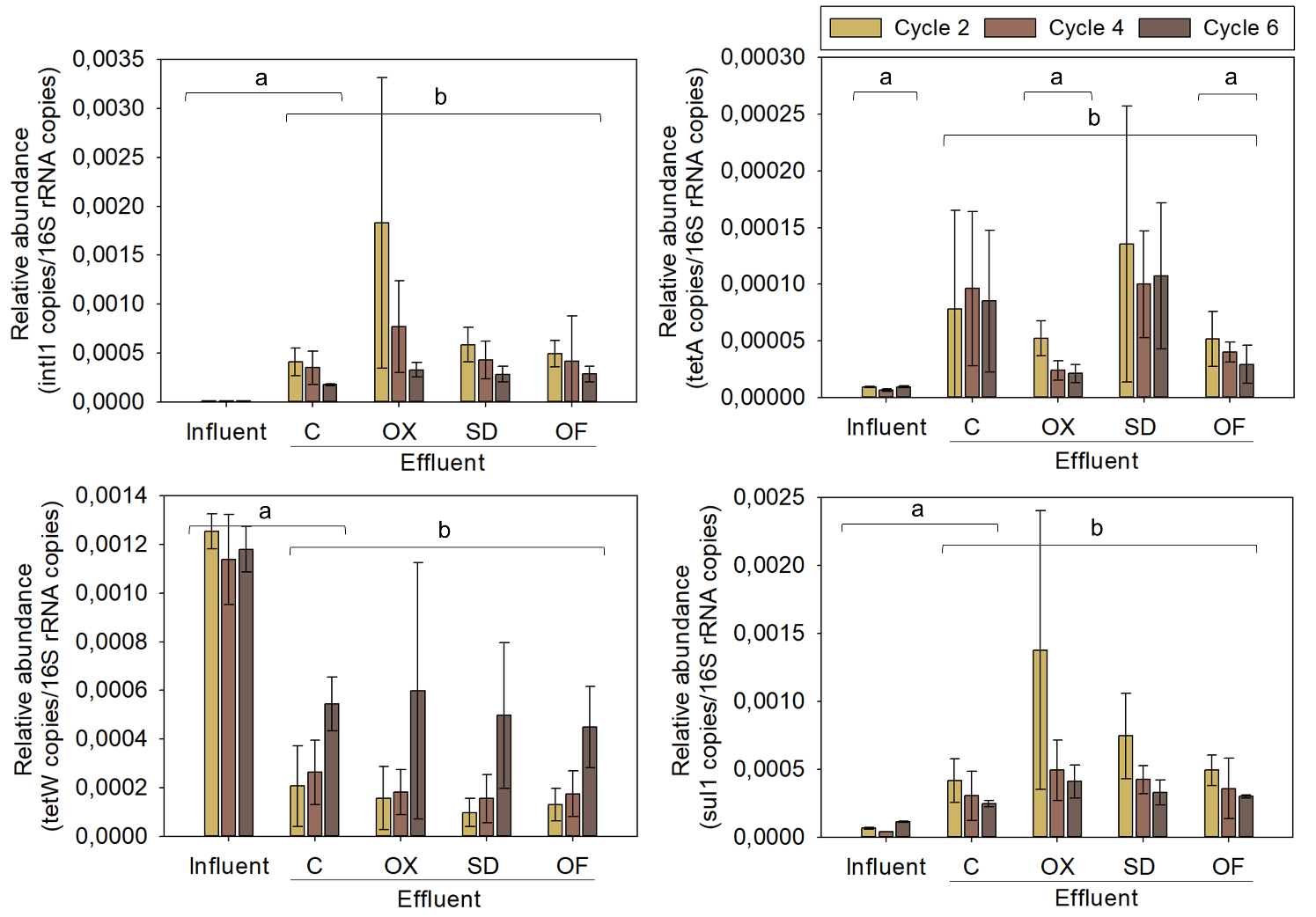
Supplementary Figure 2**. Relative abundance of ARGs in LFD before (influent, n = 6) and after (effluent, n = 36) treatment in CWs microcosms experiments. In the legend, 2, 4 and 6 indicate the number of the 14-days treatment cycle when the samples were collected. C, OX, SD, and OF correspond to the different treatment conditions in CWs (control digestate - C, digestate spiked with oxytetracycline – OX, digestate with sulfadiazine – SD and digestate with ofloxacin – OF). The letters correspond to the subsets that are not significantly different at P < 0.05 by ANOVA on ranks

| **Target genes** | **Primer sequence (5'to 3')** | **References** | **Mechanism** | **Amplicon size (bp)** | **qPCR conditions** |
| --- | --- | --- | --- | --- | --- |
| *tetA* | F: GCTACATCCTGCTTGCCTTC | Ng et al., 2001 | Efflux | **210** | 95 ºC - 10 min (1 cycle); 95 ºC - 30 seg, 60 ºC - 60 seg (40 cycles) |
|  | R: CATAGATCGCCGTGAAGAGG |  |  |  |  |
| *tetW* | F: GAGAGCCTGCTATATGCCAGC | Aminov et al., 2001 | Degradation enzyme | **168** | 95 ºC - 10 min (1 cycle); 95 ºC - 30 seg, 60 ºC - 60 seg (40 cycles) |
|  | R: GGGCGTATCCACAATGTTAAC |  |  |  |  |
| *sul1* | sul1-FW: CGCACCGGAAACATCGCTGCAC | Pei et al., 2006 | Protection | **162** | 95 ºC - 5 min (1 cycle); 95 ºC - 10 seg, 60 ºC - 30 seg (35 cycles) |
|  | sul1-RV: TGAAGTTCCGCCGCAAGGCTCG |  |  |  |  |
| *qnrS* | qnrSrtF11: GACGTGCTAACTTGCGTG | Marti & Balcázar, 2013 |  | **118** | 95 ºC - 5 min (1 cycle); 95 ºC - 15 seg, 60 ºC - 1 min (45 cycles) |
|  | qnrSrtR11 TGGCATTGTTGGAAACTT |  |  |  |  |
| *intI1* | IntI1LC5: GATCGGTCGAATGCGTGT | Barraud et al., 2010 | Class 1 integrase protein | **196** | 95 ºC - 10 min (1 cycle); 95 ºC - 15 seg, 60 ºC - 1 min (45 cycles) |
|  | IntI1LC1: GCCTTGATGTTACCCGAGAG |  |  |  |  |
| V3 region 16S rRNA | 331F: TCCTACGGGAGGCAGCAGT | Nadkarni et al., 2002 |  | **195** | 95 °C - 10 min (1 cycle); 95 °C - 15 s, 60 °C - 1 min (45 cycles) |
|  | 518R: ATTACCGCGGCTGCTGG |  |  |  |  |

Supplementary Table 1.

**Supplementary** **Table 2**. Number of reads of Illumina libraries the beginning and the end of DADA2 pipeline of the influent and effluent samples of the second, fourth and sixth treatment cycles. C, OX, SD and OF indicate the four different liquid digestates to be treated (with or without antibiotics).

| Cycle | | 2^nd^ | | 4^th^ | | 6^th^ | |
| --- | --- | --- | --- | --- | --- | --- | --- |
| Dada2 pipeline | | Input | Output | Input | Output | Input | Output |
| Influent | 1 | 93629 | 76202 | 64971 | 53308 | 60649 | 49961 |
|  | 2 | 99251 | 80125 | 72538 | 60595 | 53375 | 44319 |
| Effluent | C1 | 94818 | 63676 | 72622 | 53613 | 72439 | 53284 |
|  | C2 | 82870 | 52535 | 57200 | 40247 | 85953 | 66406 |
|  | C3 | 137947 | 97368 | 76385 | 57160 | 116214 | 92452 |
|  | OX1 | 70060 | 45205 | 78794 | 57319 | 96813 | 77543 |
|  | OX2 | 91713 | 62175 | 54521 | 38018 | 90872 | 67618 |
|  | OX3 | 94447 | 58507 | 49400 | 37736 | 119973 | 94364 |
|  | SD1 | 82599 | 55474 | 57129 | 41440 | 88797 | 68457 |
|  | SD2 | 127431 | 78684 | 88961 | 66676 | 101367 | 82028 |
|  | SD3 | 66469 | 43605 | 52875 | 34920 | 104898 | 84894 |
|  | OF1 | 121236 | 90726 | 68574 | 50003 | 112247 | 90788 |
|  | OF2 | 115008 | 79543 | 61811 | 47500 | 97730 | 80688 |
|  | OF3 | 83783 | 63127 | 66660 | 45167 | 81281 | 65152 |

|  |  |  |  |  |  |
| --- | --- | --- | --- | --- | --- |

**Supplementary Table 3.** Final concentration of COD in effluents after the treatment in CWs along the six treatment cycles. C, OX, SD and OF represent the different experimental conditions (digestate with or without antibiotics).

| **Cycle** | **Concentration of COD in the effluent (mg/L)** | | | |
| --- | --- | --- | --- | --- |
|  | **C** | **OX** | **SD** | **OF** |
| 1st | 963 ± 122 | 914 ± 124 | 1005 ± 47 | 873 ± 96 |
| 2nd | 702 ± 58 | 842 ± 150 | 944 ± 290 | 650 ± 23 |
| 3rd | 1496 ± 594 | 1129 ± 248 | 1283 ± 500 | 1367 ± 53 |
| 4th | 1965 ± 215 | 1725 ± 34 | 2086 ± 449 | 1783 ± 217 |
| 5th | 1610 ± 61 | 1647 ± 35 | 1806 ± 244 | 1531 ± 161 |
| 6th | 630 ± 146 | 560 ± 64 | 625 ± 107 | 630 ± 37 |

**Supplementary** **Table 4.** Concentration of nutrients in the effluent of CWs treating liquid digestate spiked or not with antibiotics along 6 cycles.

| **Cycle** | **Nutrient** | **Concentration of nutrients in the effluent (mg N/L or mg P/L)** | | | |
| --- | --- | --- | --- | --- | --- |
|  |  | **C** | **OX** | **SD** | **OF** |
| 1st | NH_4_^+^ | 4 ± 6 | 7 ± 12 | 1 ± 1 | 2 ± 1 |
|  | NO_3_^-^ | 1.1 ± 0.4 | 1.3 ± 0.5 | 0.8 ± 0.2 | 0.7 ± 0.4 |
|  | NO_2_^-^ | 0.1 ± 0.1 | 0.3 ± 0.3 | 0.06 ± 0.02 | 0.06 ± 0.03 |
|  | PO_4_^3-^ | 0.3 ± 0.2 | 0.24 ± 0.04 | 0.30 ± 0.06 | 0.25 ± 0.09 |
| 2nd | NH_4_^+^ | 0.9 ± 0.1 | 0.8 ± 0.2 | 1.4 ± 0.2 | 1.3 ± 0.1 |
|  | NO_3_^-^ | 1 ± 1 | 2 ± 1 | 1.1 ± 0.6 | 1.4 ± 0.5 |
|  | NO_2_^-^ | 0.05 ± 0.03 | 0.08 ± 0.08 | 0.04 ± 0.02 | 0.10 ± 0.07 |
|  | PO_4_^3-^ | 0.14 ± 0.02 | 0.15 ± 0.02 | 0.13 ± 0.03 | 0.13 ± 0.03 |
| 3rd | NH_4_^+^ | 0.7 ± 0.5 | 0.6 ± 0.6 | 0.6 ± 0.4 | 1.2 ± 0.5 |
|  | NO_3_^-^ | 3.5 ± 1 | 3.6 ± 3 | 3.1 ± 2 | 5.4 ± 3 |
|  | NO_2_^-^ | 0.12 ± 0.05 | 0.3 ± 0.3 | 0.18 ± 0.09 | 0.2 ± 0.2 |
|  | PO_4_^3-^ | 0.3 ± 0.1 | 0.17 ± 0.05 | 0.20 ± 0.07 | 0.22 ± 0.02 |
| 4th | NH_4_^+^ | 11 ± 16 | 4 ± 5 | 6 ± 3 | 16 ± 9 |
|  | NO_3_^-^ | 1 ± 1 | 2 ± 2 | 1 ± 1 | 5 ± 3 |
|  | NO_2_^-^ | 0.09 ± 0.06 | 0.06 ± 0.04 | 0.06 ± 0.03 | 0.11 ± 0.04 |
|  | PO_4_^3-^ | 0.3 ± 0.1 | 0.17 ± 0.09 | 0.20 ± 0.09 | 0.26 ± 0.06 |
| 5th | NH_4_^+^ | 12 ± 12 | 4 ± 4 | 8 ± 4 | 15 ± 6 |
|  | NO_3_^-^ | 1.1 ± 0.3 | 1.0 ± 0.1 | 3 ± 3 | 7 ± 3 |
|  | NO_2_^-^ | 0.06± 0.02 | 0.11 ± 0.03 | 0.06 ± 0.01 | 0.2 ± 0.2 |
|  | PO_4_^3-^ | 0.2 ± 0.1 | 0.28 ± 0.05 | 0.2 ± 0.1 | 0.19 ± 0.07 |
| 6th | NH_4_^+^ | 8 ± 3 | 6 ± 2 | 4 ± 1 | 9.77 ± 0.08 |
|  | NO_3_^-^ | 5 ± 2 | 5 ± 3 | 4.0 ± 0.6 | 6 ± 2 |
|  | NO_2_^-^ | 0.11 ± 0.03 | 0.14 ± 0.07 | 0.2 ± 0.2 | 0.5 ± 0.3 |
|  | PO_4_^3-^ | 0.18 ± 0.07 | 0.13 ± 0.02 | 0.21 ± 0.07 | 0.134 ± 0.005 |

**Supplementary** **Table 5.** Concentration of trace metals in the effluent of CWs treating liquid digestate during 6 treatment cycles. <LOD indicates that the concentration is below the limit of detection.

| **Cycle** | **Metal** | **Concentration of metal in the effluent (mg/L)** | | | |
| --- | --- | --- | --- | --- | --- |
|  |  | **C** | **OX** | **SD** | **OF** |
| 1^st^ | Fe | 5 ± 2 | 5 ± 1 | 5 ± 2 | 5 ± 2 |
|  | Mn | 0.2 ± 0.1 | 0.15 ± 0.09 | 0.14 ± 0.07 | 0.12 ± 0.02 |
|  | Zn | 0.07 ± 0.05 | 0.03 ± 0.01 | 0.06 ± 0.03 | 0.04 ± 0.01 |
|  | Cu | < LOD | < LOD | < LOD | < LOD |
|  | Pb | 0.004 ± 0.003 | 0.0043 ± 0.0007 | 0.006 ± 0.004 | 0.003 ± 0.002 |
|  | Cr | 0.0040 ± 0.0009 | 0.0035 ± 0.0003 | 0.0039 ± 0.0003 | 0.0034 ± 0.0003 |
| 2^nd^ | Fe | 2 ± 2 | 2 ± 1 | 3 ± 1 | 2.2 ± 0.4 |
|  | Mn | 0.12 ± 0.03 | 0.11 ± 0.02 | 0.103 ± 0.006 | 0.11 ± 0.01 |
|  | Zn | < LOD | < LOD | < LOD | < LOD |
|  | Cu | < LOD | < LOD | < LOD | < LOD |
|  | Pb | 0.004 ± 0.002 | 0.0030 ± 0.0005 | 0.0031 ± 0.0004 | 0.0028 ± 0.0004 |
|  | Cr | 0.0031 ± 0.0005 | 0.0037 ± 0.0005 | 0.004 ± 0.001 | 0.0028 ± 0.0002 |
| 3^rd^ | Fe | 5 ± 2 | 3.4 ± 0.9 | 4 ± 2 | 6 ± 2 |
|  | Mn | 0.14 ± 0.07 | < LOD | < LOD | 0.15 ± 0.06 |
|  | Zn | < LOD | < LOD | < LOD | < LOD |
|  | Cu | < LOD | < LOD | < LOD | < LOD |
|  | Pb | 0.004 ± 0.002 | 0.0035 ± 0.0006 | 0.0041 ± 0.0005 | 0.0032 ± 0.0005 |
|  | Cr | 0.006 ± 0.003 | 0.004 ± 0.001 | 0.004 ± 0.001 | 0.0056 ± 0.0007 |
| 4^th^ | Fe | 7 ± 2 | 8 ± 5 | 9 ± 5 | 4.6 ± 0.9 |
|  | Mn | 0.15 ± 0.04 | 0.12 ± 0.02 | 0.11 ± 0.02 | 0.14 ± 0.05 |
|  | Zn | < LOD | < LOD | < LOD | < LOD |
|  | Cu | < LOD | < LOD | < LOD | < LOD |
|  | Pb | 0.005 ± 0.001 | 0.006 ± 0.002 | 0.006 ± 0.004 | 0.004 ± 0.002 |
|  | Cr | 0.0041 ± 0.0004 | 0.0043 ± 0.0008 | 0.0043 ± 0.0009 | 0.0043 ± 0.0001 |
| 5^th^ | Fe | 6 ± 2 | 5 ± 1 | 6 ± 2 | 3.4 ± 0.8 |
|  | Mn | 0.17 ± 0.03 | 0.17 ± 0.02 | 0.15 ± 0.06 | 0.14 ± 0.03 |
|  | Zn | 0.04 ± 0.03 | 0.06 ± 0.07 | 0.06 ± 0.06 | < LOD |
|  | Cu | 0.07 ± 0.01 | 0.08 ± 0.01 | 0.09 ± 0.03 | 0.07 ± 0.01 |
|  | Pb | 0.0077 ± 0.0008 | 0.012 ± 0.006 | 0.012 ± 0.009 | 0.0027 ± 0.0006 |
|  | Cr | 0.007 ± 0.001 | 0.0061 ± 0.0006 | 0.008 ± 0.001 | 0.0061 ± 0.0005 |
| 6^th^ | Fe | 1.9 ± 0.8 | 1.5 ± 0.6 | 2 ± 1 | 1.4 ± 0.2 |
|  | Mn | 0.12 ± 0.04 | < LOD | 0.11 ± 0.02 | 0.104 ± 0.006 |
|  | Zn | < LOD | < LOD | < LOD | < LOD |
|  | Cu | < LOD | 0.053 ± 0.005 | 0.056 ± 0.005 | 0.062 ± 0.006 |
|  | Pb | 0.005 ± 0.004 | 0.003 ± 0.002 | 0.004 ± 0.001 | 0.0028 ± 0.0002 |
|  | Cr | 0.009 ± 0.001 | 0.006 ± 0.001 | 0.0056 ± 0.0009 | 0.0058 ± 0.0006 |

**Supplementary** **Table 6.** Average relative abundance of *Clostridium* and *Streptococcus* genera in CWs treating in parallel the 4 different LFDs during the second, the fourth and the sixth 14-day treatment cycles. Abbreviations: C, control digestate; OX, digestate doped with oxytetracycline; SD, digestate doped with sulfadiazine; OF, digestate with ofloxacin; n.d., not detected.

| **Cycle** | **Genus** | **Relative abundance (%)** | | | | |
| --- | --- | --- | --- | --- | --- | --- |
|  |  | **Influent** | **Effluent** | | | |
|  |  |  | **C** | **OX** | **SD** | **OF** |
| 2^nd^ | *Clostridium_sensu_stricto_1* | 0.07 ± 0.01 | 0.03 ± 0.02 | 0.05 ± 0.04 | 0.03 ± 0.04 | 0.04 ± 0.03 |
|  | *Clostridium_sensu_stricto_15* | 0.5 ± 0.2 | 0.02 ± 0.02 | n. d. | 0.01 ± 0.01 | 0.00 ± 0.01 |
|  | *Clostridium_sensu_stricto_8* | 1.0 ± 0.2 | 0.01 ± 0.01 | n. d. | 0.01 ± 0.03 | 0.01 ± 0.01 |
|  | ∑ *Clostridium* | 1.5 ± 0.4 | 0.05 ± 0.03 | 0.05 ± 0.04 | 0.05 ± 0.05 | 0.05 ± 0.03 |
|  | *Streptococcus* | 7.9 ± 0.2 | 0.7 ± 0.6 | 0.3 ± 0.2 | 0.5 ± 0.4 | 0.6 ± 0.3 |
| 4^th^ | *Clostridium_sensu_stricto_1* | 0.12 ± 0.02 | 0.01 ± 0.01 | 0.01 ± 0.01 | 0.02 ± 0.01 | n. d. |
|  | *Clostridium_sensu_stricto_15* | 0.50 ± 0.06 | n. d. | n. d. | 0.002 + 0.004 | 0.002 ± 0.004 |
|  | *Clostridium_sensu_stricto_8* | 0.090 ± 0.004 | n. d. | n. d. | n. d. | n. d. |
|  | ∑ *Clostridium* | 0.71 ± 0.05 | 0.01 ± 0.01 | 0.01 ± 0.01 | 0.02 ± 0.02 | 0.002 ± 0.004 |
|  | *Streptococcus* | 8.3 ± 0.8 | 0.4 ± 0.1 | 0.4 ± 0.3 | 0.3 ± 0.1 | 0.3 ± 0.2 |
| 6^th^ | *Clostridium_sensu_stricto_1* | 0.158 ± 0.004 | 0.01 ± 0.01 | 0.009 ± 0.003 | 0.01 ± 0.01 | 0.01 ± 0.01 |
|  | *Clostridium_sensu_stricto_15* | 0.6 ± 0.1 | 0.02 ± 0.03 | 0.05 ± 0.01 | 0.02 ± 0.01 | 0.02 ± 0.01 |
|  | *Clostridium_sensu_stricto_8* | 0.5 ± 0.1 | 0.02 ± 0.01 | 0.05 ± 0.02 | 0.03 ± 0.02 | 0.06 ± 0.04 |
|  | ∑ *Clostridium* | 1.275 ± 0.002 | 0.05 ± 0.03 | 0.11 ± 0.02 | 0.06 ± 0.02 | 0.09 ± 0.04 |
|  | *Streptococcus* | 9 ± 1 | 1.0 ± 0.3 | 1.3 ± 0.5 | 1.2 ± 0.4 | 1.4 ± 0.1 |

**References**

Aminov, R. I., Garrigues-Jeanjean, N., & Mackie, R. I. (2001). Molecular ecology of tetracycline resistance: Development and validation of primers for detection of tetracycline resistance genes encoding ribosomal protection proteins. *Applied and Environmental Microbiology*, *67*(1), 22–32. https://doi.org/10.1128/AEM.67.1.22-32.2001

Barraud, O., Baclet, M. C., Denis, F., & Ploy, M. C. (2010). Quantitative multiplex real-time PCR for detecting class 1, 2 and 3 integrons. *Journal of Antimicrobial Chemotherapy*, *65*(8), 1642–1645. https://doi.org/10.1093/jac/dkq167

Marti, E., & Balcázar, J. L. (2013). Real-time PCR assays for quantification of qnr genes in environmental water samples and chicken feces. *Applied and Environmental Microbiology*, *79*(5), 1743–1745. https://doi.org/10.1128/AEM.03409-12

Nadkarni, M. A., Martin, F. E., Jacques, N. A., & Hunter, N. (2002). Determination of bacterial load by real-time PCR using a broad-range (universal) probe and primers set. In *Microbiology* (Vol. 148). www.angis.org.au

Ng, L. K., Martin, I., Alfa, M., & Mulvey, M. (2001). Multiplex PCR for the detection of tetracycline resistant genes. *Molecular and Cellular Probes*, *15*(4), 209–215. https://doi.org/10.1006/mcpr.2001.0363

Pei, R., Kim, S. C., Carlson, K. H., & Pruden, A. (2006). Effect of River Landscape on the sediment concentrations of antibiotics and corresponding antibiotic resistance genes (ARG). *Water Research*, *40*(12), 2427–2435. https://doi.org/10.1016/j.watres.2006.04.017
